# Assessment of stress in adolescent idiopathic scoliosis patients while wearing a brace

**DOI:** 10.1186/1748-7161-7-S1-O4

**Published:** 2012-01-27

**Authors:** T Kuru, H Yilmaz

**Affiliations:** 1 Istanbul University, Faculty of Health Sciences, Department of Physiotherapy and Rehabilitation, Istanbul, Turkey; 2 Canakkale Onsekiz Mart University, Canakkale, Turkey

## Background

The adolescent years are among the most stressful times in a person’s life [1] and if they have adolescent idiopathic scoliosis (AIS), stress response can be roused by spinal deformity and increased by brace treatment. The purpose of this study is to assess stress of patients with AIS who wear a spinal brace with the “Bad Sobernheim Stress Questionnaire (BSSQ)” [2-5].

## Materials and methods

Patients fulfilling that criteria were included this study 8-17 years of age, diagnosed with AIS, at least two months of experience wearing the brace. The BSSQ consists of 8 Likert-scale items. A maximum score of 24 indicates the lowest level of stress.

## Results

38 patients (mean age= 13.96±3.48; range 8-17) who had came Formed Physical Therapy and Rehabilitation Clinic between March 2008 and November 2010 were included in this study and fulfilled the questionnaire. They were using Cheneau brace for 11.65±11.37 months (range 2-30) 7 were male and 31 were female. Mean Cobb angle was 34.45° ± 10.89 (range 18-57) and mean rotation angle was 6.13° ± 4.19 (range 0-22). The average stress value was 11.57 ± 5.25 (range 22-3). There was a negative correlation between brace using time and stress (r= -0,870, p=0,03); and there was no correlation between age and stress (r=0,208, p=0,64).

## Conclusions

Previous studies suggested that the BSS questionnaire is reliable for deformity related stress. We used the BSSQ and our study showed that braces in AIS treatment seem to produce stress.
